# Direct implantation of hair-follicle-associated pluripotent (HAP) stem cells repairs intracerebral hemorrhage and reduces neuroinflammation in mouse model

**DOI:** 10.1371/journal.pone.0280304

**Published:** 2023-01-13

**Authors:** Koya Obara, Kyoumi Shirai, Yuko Hamada, Nobuko Arakawa, Ayami Hasegawa, Nanako Takaoka, Ryoichi Aki, Robert M. Hoffman, Yasuyuki Amoh

**Affiliations:** 1 Department of Dermatology, Kitasato University School of Medicine, Sagamihara, Kanagawa, Japan; 2 AntiCancer, Inc., San Diego, California, United States of America; 3 Department of Surgery, University of California San Diego, San Diego, California, United States of America; University of Minnesota Medical School, UNITED STATES

## Abstract

Intracerebral hemorrhage (ICH) is a leading cause of mortality with ineffective treatment. Hair-follicle-associated pluripotent (HAP) stem cells can differentiate into neurons, glial cells and many other types of cells. HAP stem cells have been shown to repair peripheral-nerve and spinal-cord injury in mouse models. In the present study, HAP stem cells from C57BL/6J mice were implanted into the injured brain of C57BL/6J or nude mice with induced ICH. After allo transplantation, HAP stem cells differentiated to neurons, astrocytes, oligodendrocytes, and microglia in the ICH site of nude mice. After autologous transplantation in C57BL/6J mice, HAP stem cells suppressed astrocyte and microglia infiltration in the injured brain. The mRNA expression levels of IL-10 and TGF-β1, measured by quantitative Real-Time RT-PCR, in the brain of C57BL/6J mice with ICH was increased by HAP-stem-cell implantation compared to the non-implanted mice. Quantitative sensorimotor function analysis, with modified limb-placing test and the cylinder test, demonstrated a significant functional improvement in the HAP-stem-cell-implanted C57BL/6J mice, compared to non-implanted mice. HAP stem cells have critical advantages over induced pluripotent stem cells, embryonic stem cells as they do not develop tumors, are autologous, and do not require genetic manipulation. The present study demonstrates future clinical potential of HAP-stem-cell repair of ICH, currently a recalcitrant disease.

## Introduction

About 15% of acute strokes are caused by intracerebral hemorrhage (ICH), which has a death rate of 45% within 30 days and leaves the majority of survivors with neurological impairment [1,2]. Despite many therapeutic options, survivors often experience significant, long-lasting neurologic damage [3]. The main causes of primary brain damage after ICH are the initial hemorrhage and subsequent hematoma expansion. The presence of parenchymal blood causes secondary brain damage, which worsens the prognosis for patients [4,5]. Excitotoxicity, edema, and the lysis of erythrocytes that releases their iron content contribute to the development of secondary brain damage. Ferrous iron causes the production of reactive oxygen species, which is neurotoxic [6]. Around the ICH, inflammation caused by leucocyte infiltration, astrocyte stimulation, and microglia activation results in cell death from apoptosis [7–9]. Currently, surgery, managing intracranial hypertension and blood pressure, reducing cerebral edema, supportive care, and rehabilitation are treatments for ICH. However, the effectiveness of therapeutic intervention is yet only partially shown [10]. Against this background, there is a growing need for new effective therapies for the treatment of ICH. Stem cell therapy, as a promising approach, has attracted great interest from researchers around the world.

Our laboratory discovered hair-follicle-associated pluripotent (HAP) stem cells, located in the bulge area [11,12], HAP stem cells from mouse and human expressed nestin and could differentiate to neurons, glia, keratinocytes, smooth-muscle cells, melanocytes and beating cardiac muscle cells in vitro [13–17]. Previously, we have presented that HAP stem cells encapsulated in polyvinylidene fluoride membranes effect the severed sciatic nerve in the mouse model and spinal cord injury in the acute and early chronic phase in the mouse model [18–22].

In the present study, we demonstrated that mouse HAP stem cells can affect structural and functional recovery of ICH in mouse models. The potential clinical advantages of HAP stem cells for ICH therapy are discussed.

## Materials and methods

### Animals

Transgenic C57BL/6J-EGFPmice (GFP mice) were purchased from the Research Institute for Microbial Diseases at Osaka University in Osaka, Japan [23]. C57BL/6J female mice and BALB/cAJcl-nu/nu female mice (nude mice) were purchased from CLEA Japan (Tokyo, Japan). The experimental animals were housed in a system that kept the temperature at 24±1°C, relative humidity at 50–60%, and the light-dark cycle at 14 hours and 10 hours, respectively. All procedures using animals in accordance with the guidelines of the US National Institutes of Health and were approved by the Animal Experimentation and Ethics Committees of the Kitasato University School of Medicine (No. 2021–024). All mouse experiments were in accordance with animal welfare laws, complied with ARRIVE guidelines. The method of euthanasia at the end of the experiment was cervical dislocation.

### Isolation and culture of HAP stem cells

**Fig 1:**
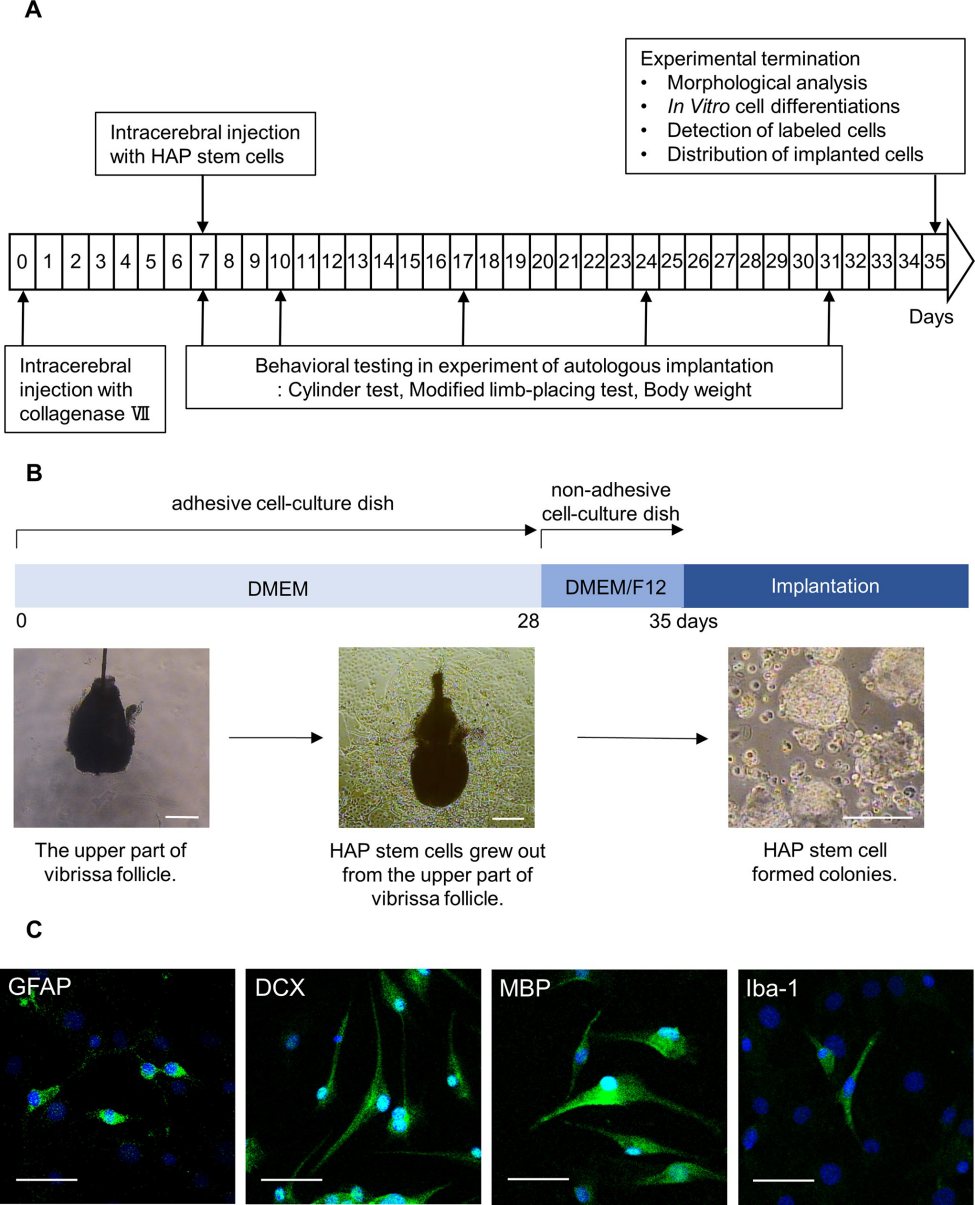
Experimental flow diagram and culture of HAP stem cells. (B) Procedure for culture and colonization of HAP stem cells. Bar = 200 μm. (C) Immunofluorescence staining shows that the cultured HAP stem cell colonies differentiated to GFAP-positive astrocytes, DCX-positive neurons, MBP-positive oligodendrocytes and Iba-1-positive microglia on Lab-Tek chamber slides. Green = GFAP, DCX, MBP or Iba-1; Blue = DAPI. Bar = 50 μm.

The schema of the present experimental study is shown in Fig 1A. Vibrissa hair follicles were obtained from green fluorescent protein (GFP) expressing transgenic or non-GFP C57BL/6J mice as described previously [15]. The mice were anesthetized with a combination anesthetic of 0.75 mg/kg medetomidine, 4.0 mg/kg midazolam and 5.0 mg/kg butorphanol [24]. The inner surface of a vibrissa pad was exposed and vibrissa follicles were dissected under a binocular microscope. The vibrissa follicle was divided into upper and lower parts and the upper parts were cultured in DMEM (#D6429, Sigma Aldrich, St. Louis, MO, USA), containing 10% fetal bovine serum (FBS), 50 μg/ml gentamycin (#15750–060, GIBCO, Grand Island, NY, USA), 2 mM L-glutamine (#25030, GIBCO) and 10 mM HEPES (#H0887, Sigma Aldrich) for four weeks in order to grow HAP stem cells at which point they were detached and transferred to non-adhesive cell-culture dishes with DMEM/F12 (#11320–033, GIBCO), containing 2% B-27 (#17504044, GIBCO) and 5 ng/ml basic fibroblast growth factor (bFGF) (#GF003, Millipore, Temecula, CA, USA). The HAP stem cells formed colonies in one week in this model [15]. To confirm their differentiation with immunostaining, HAP stem cell colonies were cultured in Lab-Tek chamber slides (Nunc, Rochester, NY, USA) with DMEM for one weeks. No chemical additives to differentiate the HAP stem cell were used during all culture period.

### Mouse ICH model

ICH was induced by stereotaxic, intra-striatal administration of bacterial collagenase by previously described protocols with minor modifications [25–27]. In brief, we made a midline scalp incision and drilled a hole of cranium (0.2 mm anterior, 3.8 mm ventral, and 2.0 mm lateral to the bregma) under anesthesia. A solution of collagenase VII (0.075 U in 0.5 μl volume, #LS005332, Worthington Biochemical Lakewood, NJ, USA) was injected into the striatum with a micro infusion syringe with a fixed needle (3.8 mm ventral) (Hamilton 87942, Hamilton, Allston, MA, USA) from the cranial hole at a rate of 0.25 μl/min for 2 min. The needle was left in place for an additional 10 min after injection to avoid reflux. The incision was closed with 6–0 nylon sutures (SIGMA REX, Tokyo, Japan). Animals were maintained in a separate cage.

### Implantation of HAP stem cell colonies in the mouse ICH model

Nude mice or C57BL/6J mice with ICH were randomly divided into two groups, HAP-stem cell injection, and non-injected mice seven days after the induction of ICH. A midline scalp incision was made and HAP stem cells (4 × 10^4^) in PBS in a total fluid volume of 2 μl were implanted with a micro infusion syringe with a fixed needle at a rate of 1.0 μl/min for 2 min into the ICH lesion from the cranial hole and 3.8 mm ventral under anesthesia. The needle was drawn out slowly 10 min after cell implantation. The control mice received the same injection process with puncture with a needle alone, respectively. The surgical skin wound was closed with 6–0 nylon sutures. Mice that died during course of the experiment were excluded from the evaluation.

### Histological analysis

HAP stem cell colonies were cultured on Lab-Tek chamber slides, were incubated with the following antibodies: anti-glial fibrillary acidic protein (GFAP) mouse monoclonal antibody (1:200, #14-9892-82, Invitrogen, CA, USA); anti-doublecortin (DCX) mouse monoclonal antibody (1:1000, #NBP1-92684SS, Novus Biologicals Centennial, CO, USA); anti-myelin basic protein (MBP) rabbit polyclonal antibody (1:200, #AB980, Chemicon, Temecula, CA, USA) and anti-Iba-1 mouse monoclonal antibody (1:500, #012–26723, FUJIFILM Wako, Tokyo, Japan). The HAP stem cells were then incubated with goat anti-mouse IgG conjugated with Alexa Flour 488® (1:400, Molecular Probes, OR, USA), goat anti-rabbit IgG conjugated with Alexa Fluor 488® (1:400, Molecular Probes), and 4’,6-diamino-2-phenylindole, dihydrochloride (DAPI) (Molecular Probes).

At 42 days post-ICH and 35 days post-HAP-stem-cell implantation, whole brain tissue of mice was removed after mouse sacrifice by cervical dislocation. The brain tissue was divided in half at the site of the needle insertion scar coronally with a mouse brain slicer (ASI instruments Warren, MI, USA). Raw specimens of the cut surface of the nude mouse brain were directly observed with fluorescence microscopy (Stereo Microscope SZX16, Olympus, Tokyo, Japan). Afterwards, both nude mice and C57BL/6J mouse brain were formalin-fixed, and paraffin-embedded-blocks (FFPB) were made. The brain tissues were sectioned coronally at 4-μm thickness and resulting slides were stained with hematoxylin and eosin (H&E), or immunostained. The anterior and posterior brain tissue was sliced every 100 μm. A section that continued the site with the needle insertion scar was confirmed within the same area of the striatum at the level of the medial septum area, was evaluated. For immunofluorescence staining, the brain tissues were observed by fluorescence microscopy (LSM 710 microscope, Carl Zeiss, Oberkochen, Germany). FFPB sections were incubated with anti-GFAP, anti-DCX, anti-Iba-1 antibodies, and each were incubated with anti-GFP rabbit monoclonal antibody (1:1000, #600–308, Novus Biologicals, CO, USA). The anti-body-treated section then were incubated with goat anti-mouse IgG conjugated with Alexa Flour 568® (1:400, Molecular Probes), goat anti-rabbit IgG conjugated with Alexa Fluor 568® (1:400, Molecular Probes), goat anti-rabbit IgG conjugated with Alexa Fluor 488® (1:400, Molecular Probes) and DAPI (Molecular Probes). Anti-MBP treated sections were incubated with goat anti-rabbit IgG conjugated with Alexa Flour 568® (1:400, Molecular Probes) and then were incubated with GFP-Booster ATTO488 (1:200, chromotek, Planegg-Martinsried, Germany) and DAPI. For immunostaining, FFPB sections on slides were incubated with anti-GFAP and anti-Iba-1 antibodies and then were treated with EnVision Detection System / HRP, Rabbit/Mouse (DAB+) (#K5007, Dako Japan, Tokyo, Japan). The developed sections were incubated with Mayer’s hematoxylin solution. Immunostaining for GFAP and Iba-1 was then performed, in the control and implanted brain. The immunostaining intensity of GFAP- and Iba-1-positive areas were measured in three random fields of the striatum area on same coronal section (n = 4, each group); Images were captured at 200× magnification with light microscopy (BX51 microscope, OLYMPUS, Tokyo, Japan). As previously described, quantitative analyses were carried out using ImageJ software (version 1.52; National Institutes of Health, USA) [21,22]. For all analyses, the threshold values were kept at the same level.

### Quantitative Real-Time RT-PCR

The paraffin-fixed brain tissue with hemorrhagic findings observed in cross-sections was cut into 20μm section, and total 50 these sections were used for quantitative Real-Time RT-PCR per mouse. After total RNA from FFPB sections was extracted using the RNeasy Plus Mini Kit (#74134, Qiagen, Hilden, Germany), the QuantiTect® Reverse Transcription Kit (#205311, Qiagen) was used to produce cDNA, Prelude PreAmp Master Mix (#638542, TAKARA, Shiga, Japan) and pooling primers were then used to pre-amplify the cDNA. Gene expressions were normalized using GAPDH. Using a CFX96 Real-Time PCR Detection System (Bio-Rad, Hercules, CA, USA) and the Power SYBR® Green PCR Master mix (#4367659, Applied Biosystems, Waltham, MA, USA), quantitative Real-Time RT-PCR was carried out. The results were evaluated using the Delta Delta Ct method. Primer sequences were as follows: IL-10 (forward, CGGGAAGACAATAACTGCACC; reverse, CGGTTAGCAGTATGTTGTCCAGC), TGF (transforming growth factor)-β1 (forward, GAGCCCGAAGCGGACTACTA; reverse, CCCGAATGTCTGACGTATTGAAG), GAPDH (forward, AGGTCGGTGTGAACGGATTTG; reverse, TGTAGACCATGTAGTTGAGGTCA).

### Neurological score after ICH

Sensorimotor function of mice was evaluated with modified limb-placing test and the cylinder test [28,29]. These tests were performed 7 days after the induction of ICH, 3 days after HAP-stem-cell implantation and every subsequent 7 days for 30 days. In the cylinder test, mice were placed in a transparent cylinder (16.5 cm high and 9 cm in diameter) with one mirror placed behind the cylinder. The use of the forelimbs during the behavior was recorded by a camera for 5 minutes, and the video was analyzed to determine how many times each forelimb made contact with the wall. (1) The first forelimb to contact the wall was considered an independent wall placement and recorded as a “left” or “right.” (2) For lateral movements along the wall, if the left and right forelimbs contacted the wall at the same time, it was recorded as a "both." (3) If the mouse contacted the wall promptly with both forelimbs contacting first, two movements were recorded as "both" and "left and right (first contacted) forelimb independent." (4) If the mouse searched the wall laterally using both forelimbs alternately, it was recorded as a "both". The score was calculated as follows. (Right forelimb movement—Left forelimb movement)/ (Right forelimb movement + Left forelimb movement + both movements). In the modified limb placement test, mice were first held for 10 cm above a table, where the forelimb extension was observed and scored: normal stretch, 0 points; abnormal flexion, 1 point. The mouse was then placed along the edge of the table, its forelimbs hanging over the edge, and was given free movement. Forelimbs and hindlimbs were both gently pulled down, and placement and recovery were both verified. Finally, the mouse was lastly moved toward the edge of the table to check for lateral forelimb positioning. Maximum neurological deficits are indicated by a score of 5, whilst normal functioning is indicated by a score of 0.

Mouse body weight was performed 7 days after the induction of ICH, 3 days after cell implantation and every 7 days for 30 days.

### Statistical analysis

The mean ± SEM was used to express all experimental data. An unpaired the Student’s *t*-test was used to assess the differences between groups in histological analysis of sectional brain area with GFAP- and Iba-1-positive regions, and quantitative RT-PCR analysis. Two-way ANOVA followed by the Bonferroni post hoc test was used to examine the differences between groups in assessments of the cylinder test, modified limb-placing test, and body weight. A probability value of *P* ≤ 0.05 is considered significant.

## Results

### Differentiation of HAP stem cell colonies to astrocytes, neurons, oligodendrocytes and microglia in culture

HAP stem cells grew out from the upper part of vibrissa follicles during 28 days of culture in the adhesive-cell culture dishes with DMEM. HAP stem cells were then detached from the dishes and transferred to the non-adhesive cell-culture dishes with DMEM/F12. Stereomicroscopy showed that HAP stem cells became aggregated and formed colonies at 35 days of culture (Fig 1B). When HAP stem cell colonies were cultured on Lab-Tek chamber slides for another one week, immunofluorescence staining showed that the HAP stem cell colonies differentiated to astrocytes, neurons, oligodendrocytes and microglia (Fig 1C).

### HAP stem cells implanted in the ICH area in nude mice differentiated to astrocytes, neurons, oligodendrocytes and microglia

**Fig 2:**
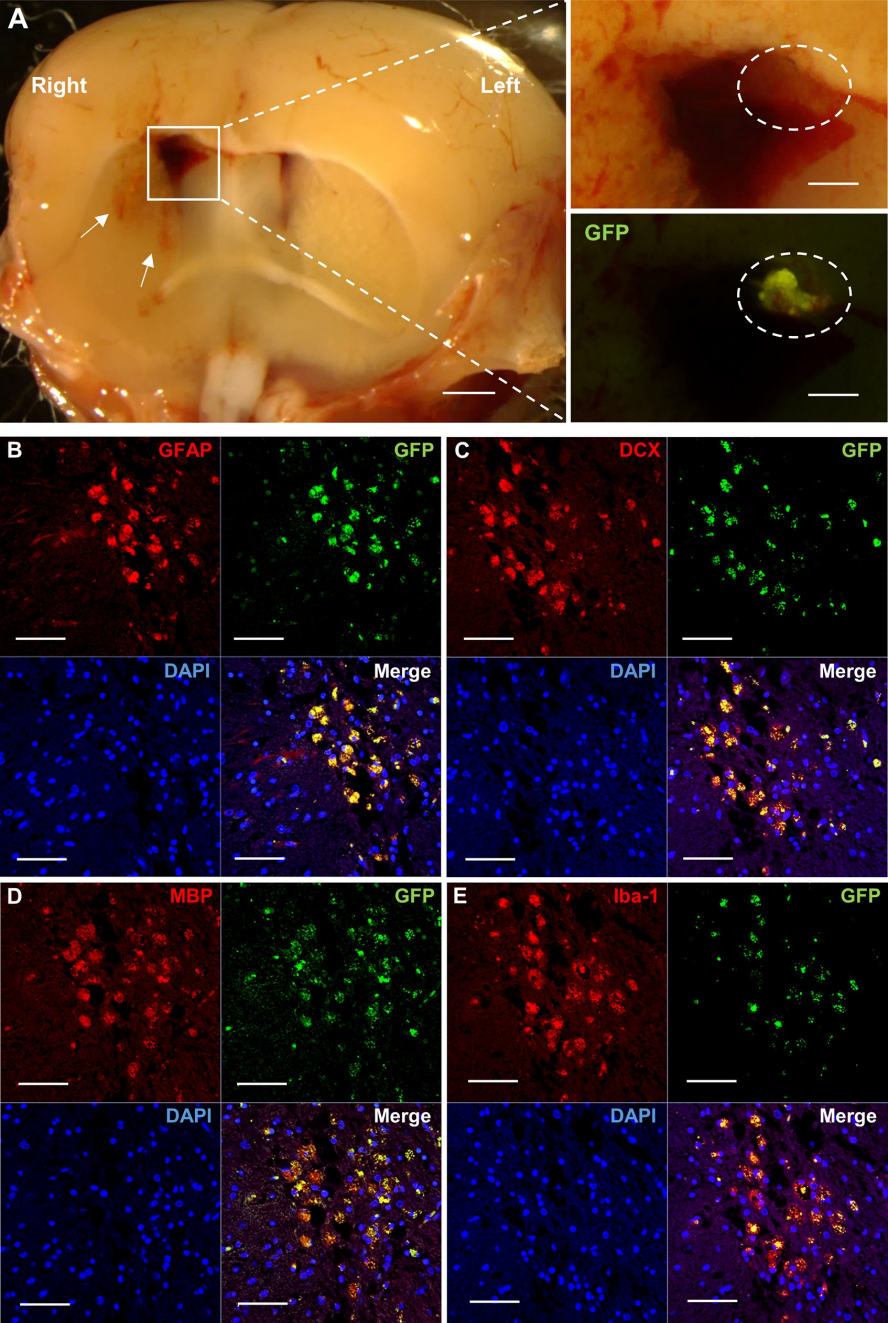
Implanted HAP stem cells repair the ICH site in nude mice. (A) Stereomicroscopy shows that GFP-expressing HAP stem cells repaired the ICH lesion. White arrow indicates hemorrhage in the striatum. White square line indicates hematoma in the brain. White dotted line indicates transplanted HAP stem cells from GFP mice in the hematoma. Left panel = low-magnification of coronal section at ICH. Bar = 1mm. Right panel = high-magnification of white square line area. Bar = 500 μm. (B-E) Immunofluorescence staining shows that the implanted HAP stem cells differentiated to astrocytes (B), neurons (C), oligodendrocytes (D) and microglia (E) in the ICH area. Red = GFAP (B), DCX (C), MBP (D) or Iba-1 (E); Green = GFP (B-E); Blue = DAPI (B-E); Merged (B-E). Bar = 50 μm. Insets show a higher magnification; Bar = 25 μm. All images show coronal sections of the brain.

HAP stem cells from GFP mice were implanted into the brain of nude mice ICH model to confirm the stem cells engraftment and differentiation. At 35 days post ICH and 28 days post-HAP-stem-cell implantation, several linear mild hemorrhages with surrounding cerebral edema in the striatum were observed in the cross section of raw nude-mouse brain specimens under the stereomicroscopy (Fig 2A). In addition, the enlargement of the right lateral ventricle and the formation of a nearby hematoma were observed (Fig 2A). The injured brain in nude mice was observed with fluorescence microscopy which showed that GFP-expressing HAP stem cells joined the damaged brain (Fig 2A). Immunofluorescence staining showed that the implanted GFP-expressing HAP stem cells differentiated into astrocytes, neurons, oligodendrocytes and microglia, which engrafted and increased in the damaged brain (Fig 2B–2E).

### HAP-stem-cell implantation blocked glial scar formation in the ICH area of C57BL/6J mice

**Fig 3:**
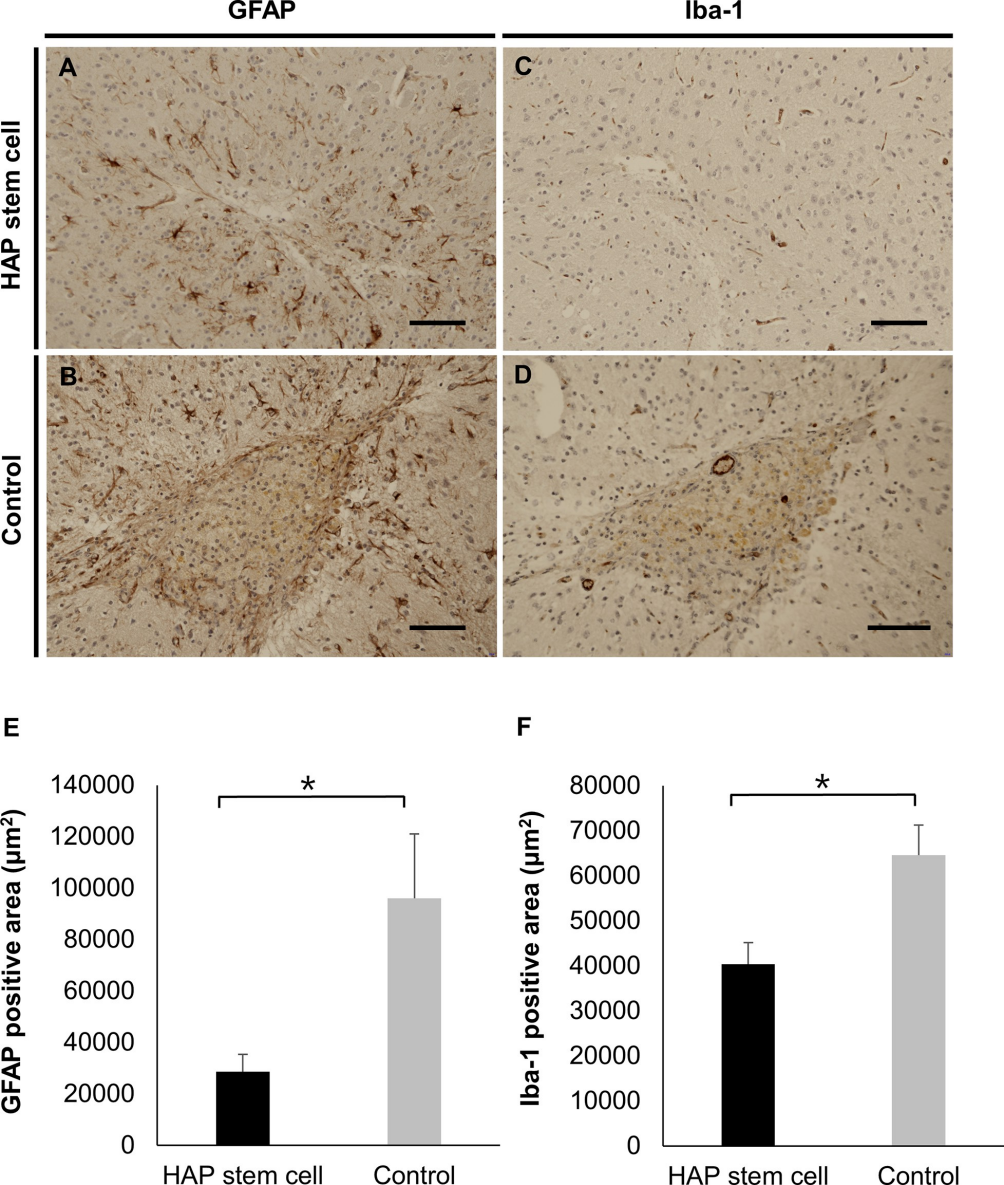
Implanted HAP stem cells inhibit glial scar formation at the ICH site in C57BL/6J. (A, B) Expression of GFAP of astrocytes in the ICH area with and without HAP-stem-cell-implantation in C57BL/6J mice. Glial scar formation was observed in the non-implanted control mice but not HAP-stem-cell-implanted mice. (C, D) Expression of Iba-1 of microglia in the ICH area with and without HAP-stem-cell implantation in C57BL/6J mice. (E) The GFAP-positive area in coronal section of the brain in C57BL/6J mice was smaller in the HAP-stem-cell-implanted mice than in the non-implanted control mice (n = 4, each group). *P < 0.05. (F) The Iba-1-positive area in coronal sections of the brain in C57BL/6J mice was smaller in the HAP-stem-cell-implanted mice than in the non-implanted control mice (n = 4, each group). *P < 0.05. Bar = 50 μm. All images show coronal sections of the brain.

HAP stem cells from C57BL/6J mice ICH model were autologous implanted into the brain to confirm the effect for neuroinflammation in the brain at 35 days post ICH and 28 days post-HAP-stem-cell implantation. Glial scar formation was observed in the non-implanted control mice and not in the HAP-stem-cell-implanted mice (Fig 3A–3D). No tumor formation was seen at the HAP-stem-cell-implanted site. There were less astrocytes demonstrated by intensity of GFAP-positive area in the ICH area in HAP-stem-cell-implanted mice than in the non-implanted control mice (*P* = 0.0111) (Fig 3E). There were also less microglia demonstrated by intensity of Iba-1-positve area in the ICH area in HAP-stem-cell-implanted mice than in the non-implanted control mice (*P* = 0.0040) (Fig 3F).

### HAP-stem-cell implantation increased the expression of IL-10 and TGF-β1 in brain of C57BL/6J mice with ICH

**Fig 4:**
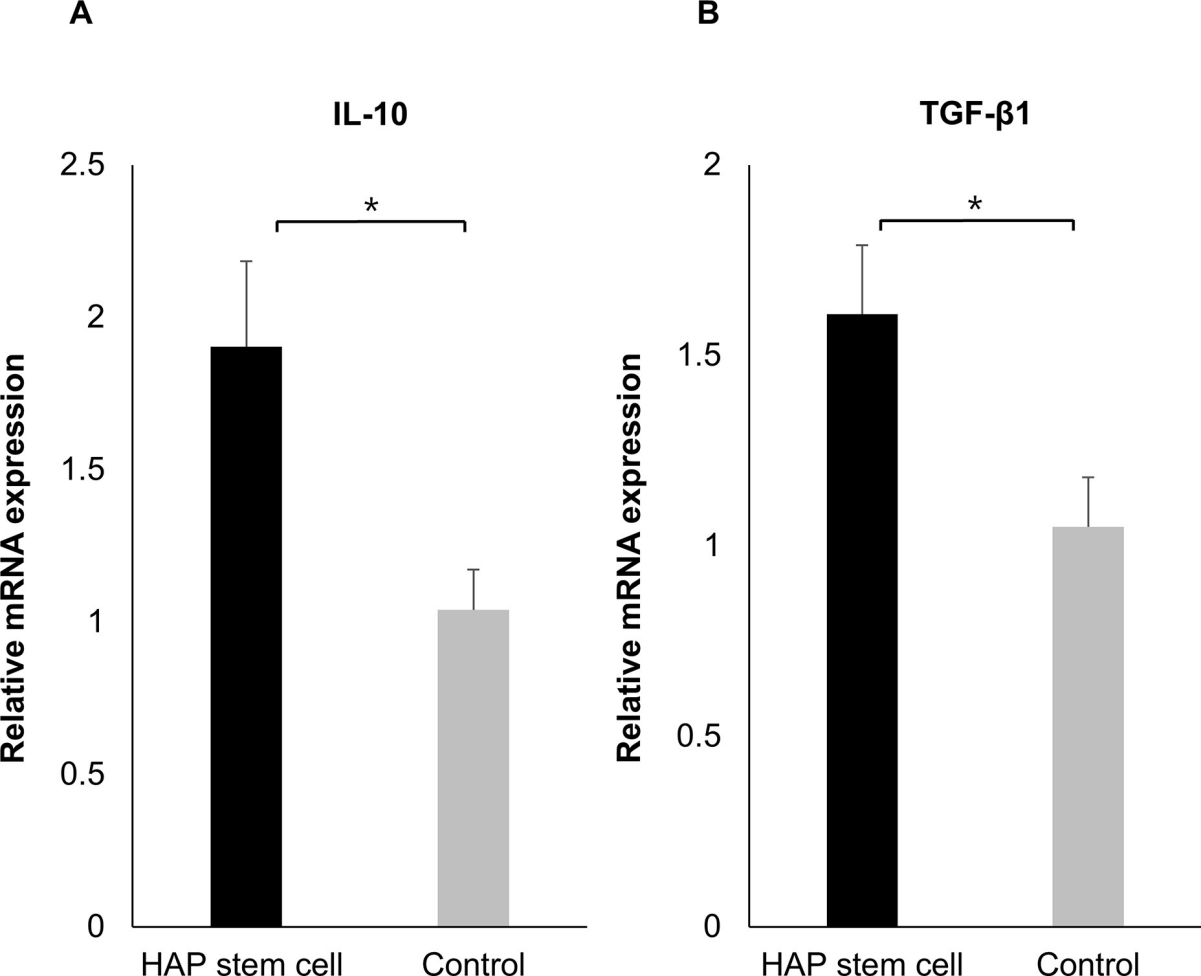
HAP-stem-cell implantation results in increased mRNA expression levels of IL-10 and TGF-β1 in C57BL/6J mice with ICH. (A) The IL-10 expression level of the brain in C57BL/6J mice was increased in the HAP-stem-cell-implanted mice compared to the non-implanted control mice (HAP-stem-cell-implanted mice: n = 12; non-implanted control mice: n = 6). *P < 0.05. (B) The TGF-β1 expression level of the brain in C57BL/6J mice was increased in the HAP-stem-cell-implanted mice compared to the non-implanted control mice (HAP-stem-cell-implanted mice: n = 12; non-implanted control mice: n = 8). *P < 0.05.

Quantitative Real-Time RT-PCR was performed using brain specimens of C57BL/6J mouse at 35 days post ICH and 28 days post-HAP-stem-cell autologous implantation to evaluate cytokines in the brain. The expression of IL-10 in the brain of C57BL/6J mice with ICH was increased compared with non-implanted control mice (P = 0.0294) (Fig 4A). The expression of TGF-β1 was also increased by HAP-stem-cell implantation compared with the non-implanted control mice (P = 0.0220) (Fig 4B).

### HAP-stem-cell implantation improves sensorimotor function and weight gain in C57BL/6J mice with ICH

**Fig 5:**
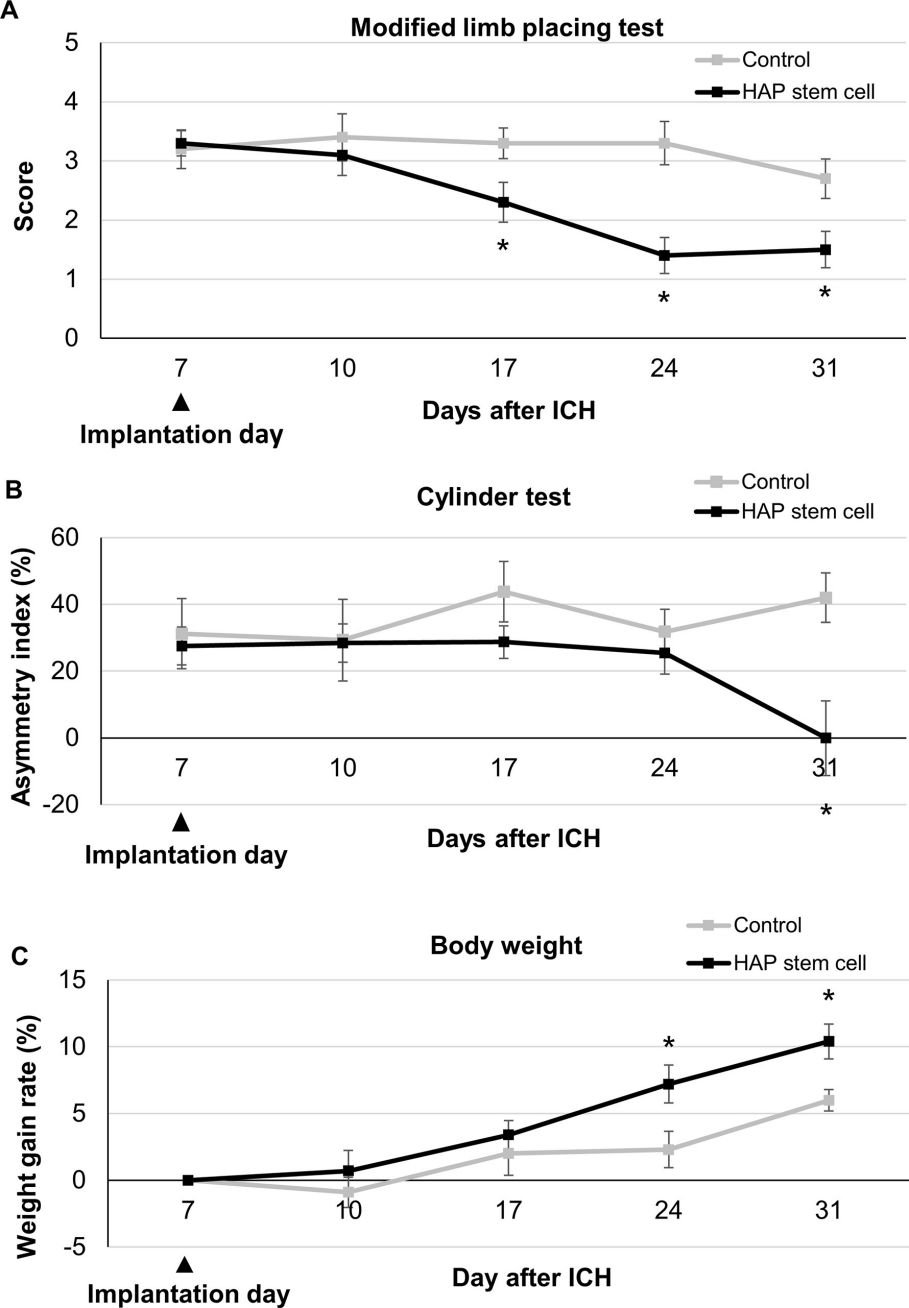
HAP stem cell implantation results in improved sensorimotor function and weight gain in C57BL/6J mice with ICH. The score of modified limb-placing test and the cylinder test and weight gain for each group over 31 days after ICH are presented. (A) C57BL/6J mice with ICH implanted with HAP stem cells had significantly better functional recovery than mice without HAP-stem-cell implantation on day 17 and thereafter in the modified limb-placing test (n = 10, each group). *P < 0.05. (B) C57BL/6J mice with ICH implanted with HAP stem cells had a significantly better functional recovery than mice without HAP-stem-cell implantation on day 31 in the cylinder test (n = 8, each group). *P < 0.05 (C) The C57BL/6J mice with ICH implanted with HAP stem cells showed significant weight gain compared to the mice without HAP-stem-cell implantation on day 24 and thereafter (n = 7, each group) *P < 0.05.

HAP stem cells from C57BL/6J mice ICH model were autologous implanted into the brain to confirm the effect for neurofunction by measuring modified limb placing test, cylinder test and body weight. The HAP-stem-cell-implanted mice showed significantly better performance in the modified limb-placing test 17 days after the induction of ICH and 10 days after HAP-stem-cell implantation, as compared with non-implanted control mice (*P* = 0.0149 at day17; *P* = 0.0004 at day 24; *P* = 0.0083 at day 31) (Fig 5A). The HAP-stem-cell implanted mice showed significantly better performance in cylinder test 31 days after the induction of ICH and 24 days after HAP-stem-cell implantation, compared with non-implanted control mice (*P* = 0.0036 at day 31) (Fig 5B). The HAP-stem-cell implanted mice showed significant weight gain 24 days after the induction of ICH and 17 days after HAP-stem-cell implantation, compared with non-implanted control mice (*P* = 0.0152 at day 24; *P* = 0.0074 at day 31) (Fig 5C).

## Discussion

It is still unclear how transplanting stem cells into animals that have suffered a stroke improves their function. Numerous processes, such as neuronal cell replacement, the production of neurotrophic factors, the encouragement of angiogenesis, the control of inflammatory response, and neuroprotection, have been postulated to explain the effects of diverse transplanted stem cells [30–33]. Induced pluripotent stem cells (iPSCs), embryonic stem cells (ESCs), neural stem cells (NSCs) and mesenchymal stem cells (MSC) have been used for ICH treatment. Qin et al. found that the transplantation of iPSCs in a rat model of ICH may have caused neuronal replacement and increased release of neurotrophic factors [34]. Nonaka et al. discovered that intracerebroventricular injections of all-trans retinoic acid-treated ESCs in a rat model of ICH, resulted in the development of neurons and glial cells around the hematoma cavity [35]. NSC transplantation has been shown in several studies to promote the functional recovery of a rodent model of ICH [26,27,36,37]. Zhou et al. reported that in a rat model of ICH, transplanted human amniotic MSCs survived slightly in the pericyte region but did not differentiate to neurons or astrocytes [38].

HAP stem cells migrate into an injured sites of peripheral nerves and spinal cord, spontaneously differentiate to cells compatible with surrounding tissue including neurons and glia and repair the injured site [18–22]. As can be seen in Fig 2 in the present study, HAP stem cells implanted into the brain 1 week after induction of ICH were found to be engrafted, despite the residual hematoma 4 weeks later. Therefore, HAP stem cells may be able to survive for a long time even in hematoma. In addition, it has been also reported that NSCs [39], MSCs [40] and Muse cells [41], which are other stem cells, can be engrafted by intracerebral injection for ICH. Under the influence of the internal environment and multiple nerve growth factors, many types of transplanted stem cells can differentiate into the two most common functional cells after ICH, neurons and glial cells; they can migrate to the injury area to replace the damaged tissues and rebuild nerve conduction pathways [42]. In the present study, allo transplantation of HAP stem cell with ICH in nude mice also differentiated into GFAP-positive astrocytes, MBP-positive oligodendrocytes, DCX-positive neuron and Iba-1-positive microglia in the injured site after ICH. Moreover, in autologous implantation of HAP stem cells in C57BL/6J mice with ICH, suppression of the glial scar formation and reactive inhibition of astrocyte infiltration and microglial infiltration were observed around the injured site, compared with the control mice. After the first ICH damage, a pro-inflammatory cascade involving activated local microglia, astrocytes, and infiltrating leucocytes results in the death of neuronal cells in the perihematomal area [7]. Chiu et al. showed that astrocytic activity inhibition improves neurologic outcomes by reducing hematoma volume and blood brain barrier (BBB) destruction [43]. In the present study, a glial scar was not formed in the HAP-stem-cell-implanted group but was formed in the non-implanted group.

In the present study, increase of the IL-10 in the brain of C57BL/6J mice implanted with HAP stem cells with ICH were observed compared with the control mice. In various neurodegenerative and neuroinflammatory condition, IL-10 is cytokine classically associated with anti-inflammatory and protective effects in the central nervous system (CNS) [44]. Villacampa et al. showed that astrocyte-targeted IL-10 production affected microglial responses and lymphocyte mobilization, ultimately having a beneficial effect on neuronal survival [45]. In the present study, increase of the TGF-β1 in the brain of C57BL/6J mice implanted with HAP stem cells with ICH were observed compared with the control mice. TGF-β1 has previously been demonstrated to play a crucial role in microglial development and homeostasis both in vitro and in vivo [46]. Tayler et al. showed that TGF-β1 enhances functional recovery in both mice and humans through regulating microglia-mediated neuroinflammation after ICH [47].

In the present study, score improvement in the modified limb placing test with HAP stem cell treatment for ICH started 10 after days HAP stem cell implantation. On the other hands, functional recovery in the cylinder test and weight gain started delayed after 24 days. Other types of stem cell treatment for ICH have been also showed both early and delayed functional recovery. Uchida et al. reported early and delayed functional recovery by Muse cell transplantation in the rat middle cerebral artery occlusion model [48]. In a rat model of ICH using collagenase, adipose-derived stem cells led to significant functional recovery after three days [49]. In a rat model of ICH with collagenase, bone marrow stromal cells led to significant functional improvement after seven days [50]. After stem cell implantation into ICH rodent model, early functional recovery within a few days may be caused by neurotrophic effect or regulation of neuroinflammation, while delayed functional recovery may be caused by neural network reconstruction [48].

The present study shows a new capability of HAP stem cells, which is repair of ICH. Direct injection of HAP stem cells into the hematoma cavity resulted in survival of implanted cells, spontaneous differentiation into neuronal marker-positive cells, suppression of glial scar formation and neuroinflammation and improvement in neurological deficit. Moreover, none of the mice implanted with HAP stem cell in the present study and previously reported studies with mice implanted with HAP stem cells into spinal cord showed any tumor formation [21,22]. HAP stem cells do not need introduction of oncogenic factors that are essential for iPSCs generation. iPSCs can form tumors unlike HAP stem cells. HAP stem cells are readily accessible from everyone, and can be used autologously without immune-suppression, do not form tumors, and can be cryopreserved without loss of pluripotency, allowing banking [51,52]. In the present study, a mouse model of ICH was used to demonstrate that HAP stem cells can be implanted in the brain of the mouse, providing beneficial effects of functional and structural recovery after ICH. We suggest HAP stem cells may have potential for repair of ICH.

## Supporting information

S1 Fig Negative control slices of GFAP (A), DCX (B), MBP (C) and Iba-1 (D) where no primary antibodies are added in Fig 2. Bar = 25 μm. All images show coronal sections of the brain.
(TIF)

S1 Table Raw data used for graphs and calculations. (XLSX)
