# Supplementary figures and images for: Direct implantation of hair-follicle-associated pluripotent (HAP) stem cells repairs intracerebral hemorrhage and reduces neuroinflammation in mouse model
Source: PLoS One. 2023 Jan 13;18(1):e0280304. doi: 10.1371/journal.pone.0280304 (PMC9838830; doi:10.1371/journal.pone.0280304)

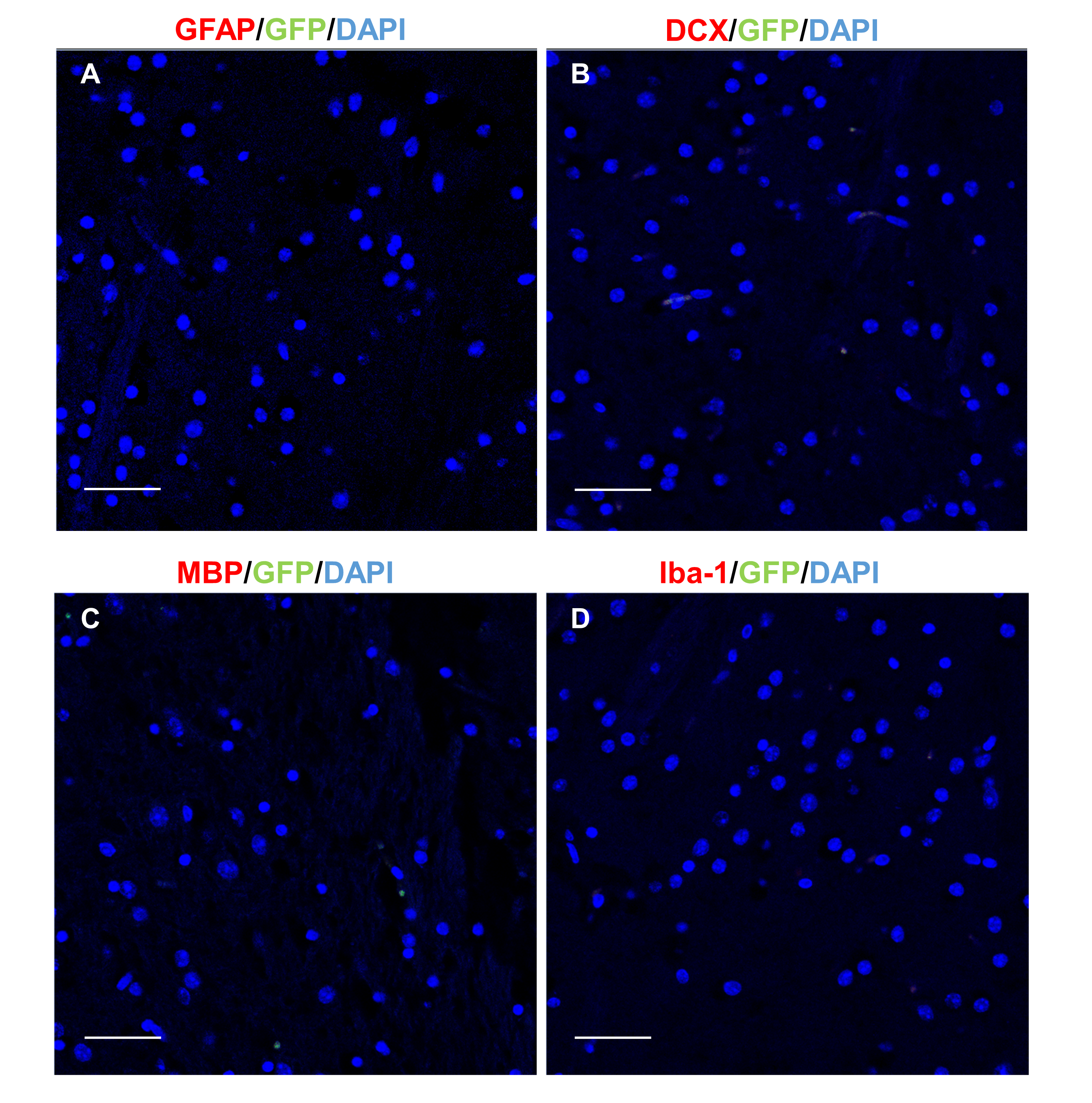

Supplement: S1 Fig — Negative control slices of GFAP (A), DCX (B), MBP (C) and Iba-1 (D) where no primary antibodies are added in Fig 2. Bar = 25 μm. All images show coronal sections of the brain. (TIF) [file pone.0280304.s001.tif]
